# Towards digitally mediated social work – the impact of the COVID-19 pandemic on encountering clients in social work

**DOI:** 10.1177/14733250221075603

**Published:** 2022-04-11

**Authors:** Vera Fiorentino, Marjo Romakkaniemi, Timo Harrikari, Sanna Saraniemi, Laura Tiitinen

**Affiliations:** 410680 University of Lapland, Rovaniemi, Finland

**Keywords:** Crisis, communication, service development, digital social work, digital space, Covid-19 pandemic

## Abstract

In the spring of 2020, the COVID-19 pandemic spread around the globe. The viral outbreak was followed by rapid changes in people’s everyday and working lives. Because of the wide-scale societal restrictions that took place to prevent the pandemic, social work was forced to take a digital leap. In this article, we examine Finnish social workers’ experiences of extending the use of digitally mediated social work (DMSW) in working with clients during the first wave of the pandemic, the spring of 2020. The data consist of 33 social workers’ personal diaries, which are analysed using a qualitative theory-based content analysis. Henri Lefebvre’s theory of spatial triad will be utilised in theorising how social workers represent DMSW through three dimensions of space, that is, how they perceive, conceive and live digital spaces when encountering their clients and how physical, mental and social spaces are embodied in the representations. The results suggest that the three dimensions of space 1) basis of, 2) conceived and 3) lived DMSW intertwine closely together. The results reveal how the physical space, including IT infrastructure, its functionality and applicability, along with the organisational contexts, form a bedrock for the social workers’ DMSW practice and had a decisive impact on their experiences. Second, the conceived space consists of workers’ cognitive and emotional elements, such as competencies, preconceptions and attitudes towards ICT. Finally, the third dimension of spatiality concludes with the social and relational aspects of the user experiences and encounters between clients and social workers.

## Introduction

The first discussions on utilising information technology in social work took place in the 1990s (Mishna et al., 2012; Reamer, 2013). Since then, digital social work has been increasingly studied and developed, but the progress in social work practices has been slow; however, there are claims of social workers being more proactive in taking full advantage of the possibilities of ICT (e.g. Craig and Calleja Lorenzo, 2014). Digital devices have potential that is not fully acknowledged (Pink et al., 2021). Here, the emerging digital possibilities have actualised the questions of ethics and risk management, which means that the stabilising of digitally mediated social work has been slow (Reamer, 2013).

The main question in using digital platforms to meet clients leads to the core of social work: how to promote social justice and inclusion in society and enhance people’s well-being and empowerment. On the one hand, there are discussions about information technology generating inequality and leading to the digital divide (Steyaert and Gould, 2009). On the other hand, debates have emerged about the harmful disjunction between social work professionals and the accelerating cultural development if ICT is not incorporated in social work. From this viewpoint, ICT has the potential to create more socially just practices (Wolf and Goldkind, 2016). In the concept of the *intraprofessional digital divide*, Wolf and Goldkind (2016) refer to a setting where some social workers are fluent with modern technologies and perceive technology-enabled opportunities more frequently than others.

Relationship-based practice has a long tradition in social work (Ruch et al., 2010). The idea of social work requiring personal presence and face-to-face contacts is still strong, and the development of digital social work has been regarded as inevitable but delayed and something that will eventually come in the future (Csoba and Diebel, 2020). In Finland, social workers’ attitudes towards digitalisation are quite positive. Social workers utilise digital platforms mainly in cooperation with collaborators and professional education or information retrieval but rarely in client work. In a recent Finnish study, 75% of the respondents had never used video connections in client work (Pyykönen, 2020).

In the current article, we will analyse social workers’ experiences of extending digitally mediated social work (DMSW) at the beginning of the COVID-19 pandemic in the spring of 2020. We will utilise *the concept of digitally mediated social work,* which refers to processing and managing information, implementing services and using social media platforms. Using the concept of DMSW, we refer to the same dimension of client work that used to be implemented face-to-face but that now must be reorganised remotely through digital devices as physical distancing between people became necessary at the beginning of the pandemic.

Moreover, we will explore DMSW by utilising the concept of spatiality. In terms of conceptualising digital space, we apply Henri Lefebvre’s (1991) theory of spatial dialectics, which enables us to study digital platforms as multidimensional social realities, including the various negotiations, articulations and lived experiences of space (Jeyasingham, 2014). Consequently, we are interested in the question of what kind of *space* digital platforms create for social work. Three specific research questions will be set: 1) How do social workers represent DMSW in the early phase of the pandemic? 2) What types of obstacles and possibilities were presented in relation to digitally mediated client work? 3) What kind of experiences did social workers gain from DMSW? The data consist of 33 personal diaries that social work professionals created from mid-March to the end of May 2020. The diaries were analysed using a thematic content analysis.

## Encountering clients in social work digitally

Since the 1990s, communication technology has been utilised in various social work practices (Mishna et al., 2012). In addition, empirical studies on performing social work on digital platforms have been increasingly conducted. Studies in the realm of social work have focused on, for example, the ethics of digitalisation (e.g. Reamer, 2017), the pros and cons of digitalisation (Craig and Calleja Lorenzo, 2014), digital competence (Zhu and Andersen, 2021), social media (Boddy and Dominelli, 2017), creating confidence or working alliances (Simpson, 2017), standardising social work and professional discretion (Philips, 2019) and the implications of digitalisation on communication (Golightley and Holloway, 2020; Mishna et al., 2012).

The research on DMSW demonstrates the complex relationship between social work and ICT. Incorporating technologies have been regarded as a ‘Pandora’s box’ comprising unexpected consequences (Mishna et al., 2012) or carrying the risk for deprofessionalisation in terms of narrowing professional judgement and the use of skills (O’Looney, 2005; Parrot and Madoc-Jones, 2008). Moreover, DMSW practices may be located in the grey zone of professional ethics because the privacy and confidentiality of clients and workers may not be ensured; hence, the questions of liability become blurred (Mishna et al., 2012). Remote working may lead to social workers missing sensory and atmospheric elements (Cook et al., 2020; Pink et al., 2020). The settings between clients and social workers are redefined, so issues such as practitioner’s availability, shared roles, professional boundaries and digital exclusion must be reconsidered.

Although the role of face-to-face meetings in creating confidential relationships with clients is important, studies suggest that the development of DMSW is inevitable and that social workers ought to be proactive in using digital devices (Parrott and Madoc-Jones, 2008; Pink et al., 2021; Simpson, 2017). Hereby, the concept of ‘social presence’ is not only confined to the scope of face-to-face contacts, but it can also occur in digitally mediated encounters. Social presence refers to the experience of being present, the degree of salience of the other person, being involved and being engaged in activities (LaMendola, 2010). In mediated conversations, affective responses, expressions of emotions or self-disclosure are the cues of social presence. Aspirations to open communication and dialogue can strengthen social presence.

Social presence intertwines closely with the concept of digital intimacy. Digital intimacy refers to intimacy that is formed in one’s digital surroundings, such as social media platforms (Pink et al., 2021). Intimacy in digital contexts is constituted within encounters where people share things that are important to them in everyday life practices. Digital encounters can generate trust because they offer service users more control in forming relationships with the social worker. Digital encounters can be more easily rescheduled or refused and can enable organising meetings with clients at the time and place best suitable for their needs. Pink et al. (2020) argue that when encountering the client, digital intimacy can be seen as beneficial as face-to-face contact, and valuable outcomes can be achieved. Digital intimacy can be created by ‘new modes of closeness, ways of sensing, understanding, care, responsiveness and support’ (Pink et al., 2020: 6).

Recently, the relation between DMSW and the COVID-19 pandemic has been a popular target of studies. Studies have focused on the resilience of social workers during the pandemic (Cook et al., 2020) and the extension of the utilisation of digital tools, which has brought new possibilities to social work and social work education (Csoba and Diebel, 2020). In general, these studies have suggested that even if there have been a lot of uncertainties regarding the usability of digital tools and information security, digital devices have been utilised in practice. In Finland, the COVID-19 pandemic forced social work to take a digital leap forward with the necessity of social distancing and working remotely. When COVID-19 hit, it became obvious that there was inadequate infrastructure and skills for enabling remote work and having competence for using digital platforms (Harrikari et al., 2021). Thus, the pandemic era has strengthened the conception that social work should be developed as a hybrid system, that is, communicating with clients both digitally and physically simultaneously (Pink et al., 2020). This raises the question of *the places and spaces* where social work is implemented.

Even if the spatial dimension is a permanent element of social analysis, the concept of *space* is not widely utilised in social work studies (Jeyasingham, 2014). While enabling an analysis of social work practices in their contexts, how spaces are constructed and represented and how space operates in social work, the spatial dimension can be used to explore an implicit background factor. Social work studies that address place usually focus on concrete, psychical places, such as homes, neighbourhoods, the mobility of social work practice (Ferguson, 2018), producing space in practice (Jeyasingham, 2014) and therapeutic spaces (Bondi, 2005).

In the present article, though, we explore DMSW as a *space*. That is, ICT enables the surroundings where social workers encounter their clients, and the space that is created is meaningful in achieving the goals of the work. Consequently, space is not any type of passive background or condition of social relations, but it is continuously produced and reproduced through social practices, relations and experiences (Jeyasingham, 2020). The spatial dimension is inextricably linked to the temporal dimension of any social action or relations: ‘spaces only exist in the sense that they occur, change and move through time’ (Jeyasingham, 2014: 1884).

While conceptualising spatial dimensions and analysing space empirically, we utilise Henri Lefevre’s (1991) conceptual framework on the spatial dimensions of the physical, mental and social, each of which are related to the other two. In his writings, Lefevre sets a perceiving, understanding and living person as the centre of his frame. The key ontological and epistemological questions of space he sets are as follows: 1) How does the space reflect and depend on the relation it has with the idea of how the space is known or understood? 2) How is the space known and intertwined with the subjective experiences of the three epistemological modes of space, which together form ‘space’?

**Figure 1.**
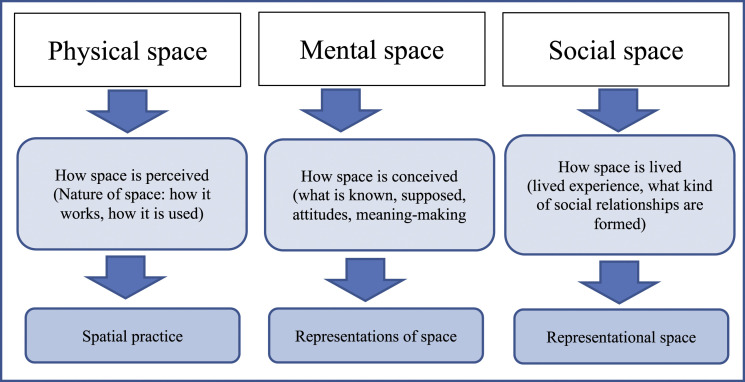
The conceptual framework of the spatial dimensions (in accordance with Hernes 2004: 81; Toyoki, 2004: 378).

In terms of analysing the spatial dimensions, Lefebvre (1991; see also Hernes, 2004; Toyoki 2004, see Figure 1) suggests a conceptual triad: spatial practise, representations of space and representational spaces. First, *spatial practice* (perceived space) embraces particular locations and the daily reality with daily routines; it ensures continuity and cohesion, and this, in turn, requires some level of competence and performance. Second, *representations of space* (conceived space) refer to the dominant spaces of knowledge, meaning, strategies, sense-making and learning. Knowledge and power are particularly present in this dimension. Finally, *representational spaces* (lived space) express the social relations that can be analysed, for example, by focussing on issues such as trust, loyalty, dependence between people and norms of behaviour.

## Methodological setting of the study: Context, data, ethics and methods

We will explore the spatial practices, representations of space and representational spaces in social work through diary data written by social workers in the spring of 2020. On 18 March 2020, the Finnish Parliament applied the Emergency Powers Act, which permitted the government to use various types of restrictions. Most restrictions took place from March to May 2020. In general, citizens were reminded of physical distancing while meeting other people. Elementary schools were closed for 2 months. High schools and universities continued operating online. Employers were instructed to order employees to work remotely from home whenever possible. Restaurants sold only takeout meals. In terms of social work, some of these services were closed, partly closed or continued providing services for the most urgent needs. Many service users who lost contact with basic social services did not receive their usual help from other services. Meetings with clients were often carried out remotely, but there were local variations (Harrikari et al., 2021).

The diary data were collected from March 15th until the end of May 2020. We regarded diaries suitable data as we aimed to receive information on social workers’ lived experiences capturing their thoughts in a chronologically organised way during this exceptional situation. According to Filep et al. (2018, 453), diary data enables to collect a ‘more nuanced understanding of everyday subjects, emotions and events’ and how these are experienced across time and space. The social workers were recruited from a closed Facebook group with more than 4000 Finnish social work professionals and focussing on the current and topical social work-related professional discussions. 33 social workers (all females, aged 30–53 years) ended up returning the diaries at the end of May, most of them writing entries every day, some week by week. The participants worked in different areas of social work, with adults, elderly care, child protection, disabled people, immigrants and addictions.

The authors were instructed to write their entries in open form recording the dates of their entries with the following questions in mind: 1) ‘What kinds of observations and experiences do you have about the phenomena and challenges that occur in the lives of social work clients during the pandemic?’; 2) ‘What challenges do social work and its practices face during a pandemic?’; and 3) ‘What kind of thoughts does the pandemic period evoke in you as a social work professional?’ The diaries were returned to the research team in digital form via secured email.

In terms of research ethics and ethical reviews, we followed the guidelines of the Finnish National Board on Research Integrity (TENK, 2019: 47–67). The participants were informed that writing a diary would be voluntary and that sending it to the research team meant giving one’s consent. The collection of background information was kept minimal: age, gender, professional title, education and the main client group they were working in at the time. We stressed that the diaries should be written as individuals and licenced social workers, not as representatives of their institutional background. If the diaries contained any identifiable information, this information was anonymised by the researchers.

The diaries were analysed using a qualitative theory-based content analysis. We first organised and reduced the data through the lenses of our research questions, that is, how DMSW is represented, what types of obstacles and possibilities are identified in implementing DMSW, what types of social practices are constructed, and the lived experiences gained. Second, we applied Lefebvre’s (1991) spatial triad as an analytical tool to explore the dimensions of space (Figure 1) with the idea that the practices of DMSW forms a space manifesting its physical, mental and social dimensions. This space can be analysed through social workers’ descriptions of how digital platforms are perceived, conceived and lived in when encountering clients (see Hernes, 2004; Toyoki, 2004) and how these descriptions embody the physical, mental and social spaces.

In our analysis, the physical dimension of *spatial practice* contains, for example, information security and ICT equipment and the know-how and rules for using them. The mental dimension, *representations of space,* includes the conceptualisations of how digital social work is understood and what kind of attitudes or thoughts are connected to it. Finally, the social dimension, *representational space,* refers to the experiences of applying DMSW and the expressions of trust and approval towards DMSW in the context of performing the core tasks of social work (see Hernes 2004; Toyoki, 2004). Based on our analysis of the diary data, we have identified three dimensions of space: 1) a basis on digitally mediated social work, 2) conceived digitally mediated social work and 3) lived digitally mediated social work.

## Basis of DMSW

The basis of DMSW consists of the infrastructure and possibilities of using it. The focus is on the resources for DMSW and the various kinds of rules and procedures that promote or insinuate the use of them. These elements form a spatial reality in which social work is implemented.

The sudden transition to remote work brought with it the slow developmental work of DMSW. Several social workers reported that the infrastructure enabling DMSW was missing or that it was out of date, here referring to such items as cameras, microphones, computers, laptops, applicable software and reliable internet connections. Obviously, the investments in infrastructure had been insufficient, leading to difficulties in applying DMSW.

*All of us do not have adequate infrastructure. We have desktops, but only a few have laptops. Only a few desktops are equipped with proper cameras or microphones. (D13/20/04/01)*

*I am trying to organise a meeting with a client on Skype, but I notice that I don’t have Skype. I try to shift the meeting to Teams, but the client doesn’t have Teams. I ended up doing a remote home visit on a phone, trying to pay attention to all four members of the family. (D6/20/03/24)*

The social workers’ descriptions related to the desktop software, mobile device applications and their data security varied. Mobile applications such as WhatsApp or Skype were forbidden in client work before the pandemic, but after the shift to remote work, in some places, they were allowed because of the lack of alternatives. The social workers did not have access to all the software they would have needed, or the new software acquired and deployed. Unusable and inappropriate ICT equipment was a nasty reality for both the social workers and their clients. In addition, the social workers were concerned about their clients’ possibilities to participate in DMSW because of a reciprocal lack of competencies between the social workers and clients at the beginning of the pandemic. Remote access work was described as frustrating. The pandemic situation forced organisations to improve their ICT infrastructure and the social workers to update their competencies:

*– – Some remote connections just don’t work or are not secure. I upload the fourth new software to my computer and learn to use it. (D10/20/04/20)*

*For most of the workers and the families that use the services, the first week was an actual digital leap. We initiated the leap by checking where one can find the camera in their computer. However, digitally mediated meetings do work well, and they definitely need to be continued after the pandemic ends. (D17/20/03/21)*

Previous studies have suggested that delays in introducing ICT possibilities in social work can be very organisational-dependent and that the utilisation of ICT strongly depends on the organisational culture and procedures, as well as management and leadership. In general, there are both implicit and explicit norms about the spatial practices in social work organisations (Jeyasingham, 2014). The organisational irregularity of ICT and evident pressure to change the spatial arrangements of work, combined with a total unpreparedness to the pandemic and lack of organisational guidelines, lead to a situation where the basic conditions for DMSW were extensively missing:

*– – All the systems of information technology either slow down or inhibit fluent client work. Sure, this matter has been brought up before, but mostly on the level of individual workers, who haven’t really been believed. Hopefully, the pandemic brings up to the public that the workers have been quite right, and someone will do something to the challenges. (D9/20/05/25)*

In the beginning of the pandemic, there was a great amount of pressure on management and superiors. In their diaries, the social workers expressed not only trust and distrust directed at their superiors and the willingness to acquire mobile devices and suitable applications, but also at the superiors’ hesitation in decision-making and delays in delivering purchases. These entries bring organisational power, leadership and boundaries into light: who is being heard, who can have digital devices, who is the one to order them and which workers are allowed to work through remote access. The functionality of infrastructure and the interplay with employees are crucial:

*I ask for a laptop so that my work would be easier, for I work at the department secretary’s computer, and if she comes back to work, I will not have any computer. The head nurse of the department states that there are no more laptops to distribute in vain and that demand for them is high at the moment. I feel that I am not heard. I swallow my disappointment and decide to try again later. I wonder, abashed, that if a doctor needs a laptop for remote work, then why can’t I have one; there is some kind of professional inequality that came forward. (D10/20/03/23)*

The diary entries of the social workers indicate how social work was obliged to be rapidly transferred to digitally mediated services in the early phase of pandemic. This change and digital space as a space for encountering social work clients led to critical debates, and the pros and cons were represented. This implies that space is an intrinsic part of social relationships, constructed as a part of social practices, relationships and experiences (Jeyasingham, 2020; Lefebvre, 1991). The question related to the availability of adequate equipment, their functionality, data security and rules clear enough for using equipment formed the space where clients’ confidentiality was established and tested. In particular, the effortless functionality of the digital connections while conducting familiar working modes and tasks emerged as the most crucial factor in the successful implementation of DMSW. The transformation of familiar digital space, which some of the social workers experienced, could be captured by the phrase ‘*earlier Skype or Teams were almost a miracle but now they are normal’ (D20/20/05/20)*. During the early phase of the pandemic, the questions of the digital divide seemed to unite the social workers (see also Wolf and Goldkind, 2016), and the rapid change generated a chaotic atmosphere and resistance. After the very beginning of the pandemic, however, DMSW was presented as a new kind of space that had crucial effects on forming client relationships. The social worker’s representations of space and technology manifest an understanding of the need for a novel kind of agency and change in the social workers’ daily routines and rhythms.

## Conceived DMSW

Conceived DMSW is associated with the question of how DMSW is signified at the very beginning and soon after it has spread as a permanent practice. The diaries make visible the social workers’ attitudes, positions, possibilities, challenges, preparedness and competencies at the starting point of the digital leap. The basis for extending the use of digital devices was manifold. Despite the general proliferation of digital devices, no systematic efforts were made to instil digital devices as a part of everyday social work. Questions such as what kind of software would be needed, how to use the software and which of the working contexts would be appropriate to use and which were not remained. Using computers consumed the precious resource of time, and many of the social workers expressed their frustration against inadequate devices and poorly functioning equipment.

In general, there was readiness among the workers to adopt digital devices into their work, but there was also resistance and a lack of competencies. In their diaries, many social workers considered how DMSW had for a long time been an option that was not fully utilised. This was explained by a lack of time, organisational investments, leadership and the reluctant attitudes of social workers. Old routines were believed to be difficult to abandon, and it felt challenging to change the existing procedures:

*I have used devices and regard them as a part of daily routines, but the same feeling is not shared among all the employees of the policlinic—resistance, with whatever excuses, can be detected (D10/20/03/20).*

*No one just has had any interest to try out this kind of new way of working, and our management has not obliged us to do it. Except now, because of the coronavirus. (D13/20/03/25)*

At the beginning of the pandemic, the competencies of both social workers and clients were vastly manifold, and the capabilities to learn how to use the devices varied broadly. The willingness to learn and use ICT was considered important, and reluctance towards using it manifested both in learning and carrying out digital meetings:

*Using remote digital connections requires willingness and competence. If the social worker is uncertain of one’s know-how or the client does not want to participate in meeting through remote access, it turns into an uncomfortable experience (D29/20/04/01).*

As mentioned, one of the key issues in extending DMSW was who, when and in what kinds of contexts meeting digitally is appropriate or not. The requirement to meet clients remotely elicited the specific importance of encountering and listening to clients. No established practices or guidelines exist for making decisions on whether to meet clients through remote access or a face-to-face format existed. Thus, the problem of DMSW is represented widely through emerging risks:

*It leads to an enormous annoyance to decide which of the clients are to be met face to face and which of them only through digital equipment. We are not willing to handle clients’ painful and emotional matters only in remote connections. Anyway, it distresses social workers that they are expected to assess risks continuously: Should I risk the client’s own or someone else’s safety or health? Should I risk the safety of a client’s child or the human encountering of the client? (D2/20/03/30)*

In their diaries, the social workers named several situations where they considered digitally mediated meetings with a client inappropriate. Addressing remarkably burdening matters and organising emotionally loaded meetings were not regarded as viable to be conducted through digital devices. Working with people with severe mental health problems (e.g. suicidal, psychotic), elderly people or people in complex life situations did not work properly through remote access. Digital meetings were considered more superficial than face-to-face encounters. In particular, the social workers were concerned about people dropping out of the services because of the digital encounters. This led them to reflect on whether they were able to recognise clients’ problems, generate profound conversations or meet a client at an emotional level in digitally mediated services. Face-to-face contact was frequently represented as a necessary condition for creating a confidential relationship with a client. After a more confidential relationship had been formed, meetings through remote access were believed to be a more conceivable option. These decisions, however, were usually made through intuition but also through professional discretion and ethical reasoning:

*Certainly, devices, software and competencies need to be okay, and the client must be willing for a remote meeting. The best guideline might be one that a social worker wrote on social media: ‘As a social worker, one can maybe only intuitively know and recognise when it is good to be present and when the matter can be handled online’. (D29/20/04/01)*

*I know that I should have handled the matter on the phone, even if there is risk that encountering a client remains on a superficial level. I feel, however, that it is my duty as a human being to meet a person when one needs it. (D3/20/03/27)*

Furthermore, the social workers described how the clients had many reasons not to come to face-to-face meetings. They mentioned situations where the remote access option was regarded as especially appropriate. Many practical issues, such as delivering information and giving advice, could be handled in digital meetings. Remote access meetings could also form a setting in which the relationship between the social worker and client was seen as more equal:

*For some, this seems to work surprisingly well: a parent can be much more active and participating when we are not in the same physical space, but they can participate in their privacy without the thrilling social setting. [--] The client can stay at home and control the situation more than in the office. This type of setting makes things more equal. (D30/20/03/27)*

In conclusion, reflections on the DMSW led the social workers to ponder over the fundamental meanings of encountering and the importance of listening. On average, a hybrid model, mixing DMSW and face-to-face encounters, was considered the most appropriate (also Pink et al., 2021). In general, face-to-face contact was regarded as necessary for successful psychosocial work. Several social workers highlighted how establishing confidential relationships with clients would require face-to-face contact, but after that, many issues can be handled through DMSW. The social workers felt left alone in an unexpected and unique pandemic setting, and they were forced to reflect on the questions of DMSW in relation to professional know-how. Promoting reciprocity, dialogue, confidentiality and feelings of safety with a client formed the cornerstones for working with clients in a pandemic setting. Professional discretion on a case-by-case basis, inclusivity and concern of exclusivity were the key issues when the social workers considered the disadvantages and benefits of DMSW.

## Lived DMSW

Within the lived DMSW, the focus became on social relations and human presence (see Toyoki, 2004). In their diaries, the social workers described their encounters and established confidence with clients, as well as the atmosphere and general conditions of the interaction. In terms of establishing relationships, concrete visibility was mentioned in the diaries as something connected to the feeling of presence and the possibility of forming a relationship, as well as making a general view of the clients’ situation. Observing clients’ own spaces, such as home, school or other parallel everyday institutions, was brought up as a crucial factor in social work with clients in a pandemic context:

*Implementing the hearing of a young person in account of taking them in care and not to have their camera on, as the young person did not like it – – was quite burdensome. It was impossible to know what was happening there in the background and it got hard for me to concentrate on the whole issue. (D17/20/04/23)*

Forming relationships and bringing difficult issues into the discussion arose as a significant matter in the diaries: ‘*Communication is not the same compared with situations where everyone is in the same place, at the same table*’ *(D19/20/03/25).* In many respects, social work is about reflecting feelings, detecting moods and debates on difficult matters, all of which had become more difficult. Several social workers expressed how they were insecure regarding the client’s real feelings while meeting them and whether all critical issues were addressed:

*I am just wondering how different clients may experience receiving services by phone. How do they feel that they are able to talk about their matters? Some of them might think that it is easier when encountering remains faceless, but some may find it more difficult just for the same reason. (D22/20/04/06)*

*In terms of the orientation and objective of social work’s orientation, it is rather confusing that we cannot meet clients face to face. Encountering and assessing a client’s situation is extremely difficult on the phone. (D22/20/03/27)*

In their diaries, the social workers frequently repeated the importance of seeing people’s faces. The elements of encountering and atmosphere in digitally mediated encounters were constantly juxtaposed and compared with ordinary live encounters with clients. The atmosphere in face-to-face meetings was described as lighter, and the recognition of faces was appraised as creating a different kind of atmosphere than in remote access meetings. Seeing faces seemed to be associated with social presence:

*Meeting the client face to face for the first time, when we had only met remotely among most of the crowd. The atmosphere is instantly somehow lighter. People don’t look the same behind cameras as they do in actual life. (D17/20/05/08)*

*During normal conditions, I would have gone and met the young client face to face just to make sure that she is okay. Now, we tried to organise video call, but emerging resistance made it impossible. Once again, I missed encountering face to face. (D26/20/04/24)*

Thus, the problem of hidden faces hampered both nonverbal interactions and verbal communication. Seeing the client offered detailed information: intuitional feelings, feelings of presence, detecting expressions and identifying gestures (also Pink et al., 2021), which promoted reciprocal dialogue. The lack of visuality was suggested as having a negative effect on interpretations; dialogue remained superficial and easily led to misunderstandings. Assessments and interpretations of the clients’ well-being were formed through many minor cues, such as presence, looks and expressions, which become more difficult in DMSW.

*Yesterday’s debate on ignoring feelings in remote access meetings continues: the employees of mother and child shelter suggest that nonverbal communication, such as countenance, gestures, sadness, crying, smiling, etc., consisting a major share of human interaction, remains ignored when meetings are conducted via remote access connections. (D2/20/05/26)*

*I met a client face to face after a long time (approximately two weeks from the previous meeting), and it felt really pleasant. It was nice to access a more dialogical and detailed communication, and I was better able to express that I care through my words and presence than on the phone. (D22/20/04/24)*

New practices were created, a new kind of language constructed and new ways of creating confidence discovered (see Pink et al., 2021). Without seeing faces, a social worker ‘*ends up describing her own appearance so that the client knows with whom he/she is talking to’ (D6/20/3/30).*

The clients that preferred digitally mediated meetings but still wanted to meet face-to-face occasionally were mentioned. The key factor for a successful encounter was the feeling of the presence of both parties. However, face-to-face meetings were seen as more suitable for social work core orientation, and the social workers expressed relief when seeing clients again.

*When meeting clients face to face, I think one can interpret a client better. Both can then specify questions and speak more naturally. I can see clients’ reactions and let myself be reachable. (D22/20/3/30)*

*But oh, how wonderful it is that there are patients in the corridor again. I have missed them! (D11/20/05/20)*

## Discussion

We have examined social workers’ experiences of extending the use of DMSW in working with clients in the context of the first wave of the COVID-19 pandemic by analysing 33 social workers’ diary entries. The results suggest that in the spring of 2020, social work was forced to take a digital leap due to the societal restrictions followed by the viral pandemic. Applying Lefebvre’s (1991) theory of the spatial triad, we captured three formations of space: 1) the basis of DMSW, 2) conceived DMSW and 3) lived DMSW (Table 1). The diaries were analysed through conceptions of how the social workers perceived, conceived and lived digital platforms when encountering their clients and how physical, mental and social spaces were embodied in these representations. However, the dimensions were not distinct but in continual dialectical relationship with each other.

**Table 1.** The spatial triad of DMSW.

Dimension	Basis of DMSW	Conceived DMSW	Lived DMSW
**Focus**	Infrastructure (devices, software, internet connections)	Competencies, preparedness using ICT	Establishing social relationships (human presence, promoting confidence, openness)
		Possibilities	
		Obstacles	
**Key factors**	Contextual functionality, functional devices, client and data security	Preconceptions, attitudes, readiness to change existing practices and learning	Atmosphere of communication, digital intimacy, reaching emotional level
**Boundary conditions**	Organisational culture (ICT training, procedures, norms, management)	Principles and discretion on using ICT	The promotion of the core aims and tasks of social work

The results reveal how *the physical space*, including IT infrastructure, its functionality and applicability, as well as the organisational contexts, helped to form a bedrock for the social workers’ DMSW practice and had a decisive impact on their experiences. Here, the physical dimension of spatiality can regulate interaction and define how space is perceived (see Hernes, 2004).

Second, *the mental space* contains the conception of ICT and its usability in client work. We focused on both these preconceptions and the cognitive and emotional elements in analysing the shift from face-to-face to digitally mediated ways of working. Moreover, the conceptions of ICT and its applicability in social work are crucial, affecting social workers’ *lived experiences*. In turn, experience can further affect the perceptions of the usage of DMSW by either confirming former presumptions or opening new possibilities. Positive experiences create pressure for improving the basis of DMSW. It seems, that in everyday practice, the three dimensions are closely intertwined and difficult to distinguish. The key question relating to DMSW and its functionality is connected to the problem of the contextual boundaries of interaction (Hernes, 2004): What kinds of boundaries are constituted between face-to-face contact and digitally mediated contacts, and how do these boundaries intersect?

Finally, the third dimension of spatiality concludes the social and relational aspects of the user experiences and encounters between clients and social workers: What kind of lived experience can the space generate, and what kind of social bonding can DMSW create? Here, the physical, mental and social dimensions are present simultaneously, but their contextual emphasis seem to vary. Even if encountering two dimensions is successful in principle, significant obstacles in the third dimension can spoil a good atmosphere when the dimensions intersect.

In our study, we examined DMSW in a transitional phase where social work was forced to shift quickly to remote access. In the middle of the crisis, a strong presumption emerged that all practices must work in the same way as before. However, digital platforms create a vastly different kind of context; many social workers faced difficulties, and it soon became obvious that the development of DMSW had been too slow. The forced digital leap made clear that DMSW is an adequate way of working and can extend options to promote the well-being of clients but holding meetings remotely under certain conditions may be more applicable than when under other conditions, forming a flexible continuum combining the best elements of face-to-face interaction and DMSW, or a ‘hybrid model’ (Pink et al., 2021). However, in the middle of the crisis that was caused by the first wave of the pandemic, the presumption that ‘everything has to be kept unchanged’ and ‘we only shift face-to-face meetings to be performed online’ limited a wide variety of functional options that DMSW could offer.

## Limitations

There are certain limitations in our analysis. The study was implemented with qualitative methods in the context of the Finnish welfare system during the first wave of the COVID-19 pandemic. When looked at from a global perspective, the infection rates have been lower in Finland than elsewhere, and the social welfare system is regarded as being good when compared against international standards. However, globally, the challenges faced by social workers seem to be quite similar. In turn, the qualitative diary data provide an authentic but limited perspective of social work practice. In addition, the social workers’ experiences and the everyday episodes described in the diaries are mediated in nature and focus on the beginning of the pandemic.

## Conclusion

In conclusion, our analysis shows how digitally mediated practices can offer promising options for extending spatial and temporal solutions to perform social work. It may be challenging to name any universal rules or guidelines for implementing DMSW because it is always a question of social workers’ discretion and expertise, too. However, the digital competence required from social workers includes technical skills and content creation, as well as the skills of enhancing dialogue and solving problems (Zhu and Andersen, 2021). As we move beyond the pandemic era, it would be appropriate to transition from the debate of individual social worker’s digital competencies or resistance towards promoting enabling conditions in DMSW as the matter of basic education and instead continuing training and working in teams and organisations.
